# A Reflection (Poetry)

**Published:** 1881-04

**Authors:** Ella Weaver


### A REFLECTION.

I fling my past behind me like a robe
Worn threadbare in the seams and out of date.
I have outgrown it. Wherefore should I weep
And dwell upon its beauty, and its dyes
Of oriental splendor ; or complain
That I must needs discard it ?

I can weave
Upon the shuttles of the future years,
A fabric far more durable.

Subdued
It may be in the blending of its hues,
Where the sombre shades commingle, yet the gleam

Of golden warp will shoot it thro' and thro'
While over all a falseless lustre lies
And starred with gems made of crystalled tears,
My new robe shall be richer than the old.

—*Ella Weaver.*

